# Oncogenic and Stemness Signatures of the High-Risk HCMV Strains in Breast Cancer Progression

**DOI:** 10.3390/cancers14174271

**Published:** 2022-09-01

**Authors:** Ranim El Baba, Sébastien Pasquereau, Sandy Haidar Ahmad, Mona Diab-Assaf, Georges Herbein

**Affiliations:** 1 Pathogens & Inflammation/EPILAB Laboratory, EA 4266, Université de Franche-Comté, Université Bourgogne Franche-Comté (UBFC), 25030 Besançon, France; 2 Molecular Cancer and Pharmaceutical Biology Laboratory, Lebanese University, Beirut 1500, Lebanon; 3 Department of Virology, CHU Besançon, 25030 Besançon, France

**Keywords:** human cytomegalovirus, CTH cells, PGCCs, HCMV strains, TNBC, paclitaxel, ganciclovir, oncogenesis

## Abstract

**Simple Summary:**

Lately, human cytomegalovirus (HCMV) has been progressively implicated in carcinogenesis alongside its oncomodulatory impact. CMV-Transformed Human mammary epithelial cells (CTH) phenotype might be defined by giant cell cycling, whereby the generation of polyploid giant cancer cells (PGCCs) could expedite the acquisition of malignant phenotypes. Herein, the main study objectives were to assess the transformation potential in vitro and evaluate the obtained cellular phenotype, the genetic and molecular features, and the activation of cellular stemness programs of HCMV strains, B544 and B693, which were previously isolated from triple-negative breast cancer (TNBC) biopsies. The strains’ sensitivity to paclitaxel and ganciclovir combination therapy was evaluated. A unique molecular landscape was unveiled in the tumor microenvironment of TNBC harboring high-risk HCMV. Overall, the explicit oncogenic and stemness signatures highlight HCMV potential in breast cancer progression thus paving the way for targeted therapies and clinical interventions which prolong the overall survival of breast cancer patients.

**Abstract:**

Background: Human cytomegalovirus (HCMV) oncomodulation, molecular mechanisms, and ability to support polyploid giant cancer cells (PGCCs) generation might underscore its contribution to oncogenesis, especially breast cancers. The heterogeneity of strains can be linked to distinct properties influencing the virus-transforming potential, cancer types induced, and patient’s clinical outcomes. Methods: We evaluated the transforming potential in vitro and assessed the acquired cellular phenotype, genetic and molecular features, and stimulation of stemness of HCMV strains, B544 and B693, isolated from EZH2^High^Myc^High^ triple-negative breast cancer (TNBC) biopsies. Therapeutic response assessment after paclitaxel (PTX) and ganciclovir (GCV) treatment was conducted in addition to the molecular characterization of the tumor microenvironment (TME). Findings: HCMV-B544 and B693 transformed human mammary epithelial cells (HMECs). We detected multinucleated and lipid droplet-filled PGCCs harboring HCMV. Colony formation was detected and Myc was overexpressed in CMV-Transformed-HMECs (CTH cells). CTH-B544 and B693 stimulated stemness and established an epithelial/mesenchymal hybrid state. HCMV-IE1 was detected in CTH long-term cultures indicating a sustained viral replication. Biopsy B693 unveiled a tumor signature predicting a poor prognosis. CTH-B544 cells were shown to be more sensitive to PTX/GCV therapy. Conclusion: The oncogenic and stemness signatures of HCMV strains accentuate the oncogenic potential of HCMV in breast cancer progression thereby leading the way for targeted therapies and innovative clinical interventions that will improve the overall survival of breast cancer patients.

## 1. Introduction

Globally, breast cancer poses a formidable health challenge as it is the most prevalent malignancy accounting for a high number of cancer deaths in women [1]. Breast cancer consists of a group of heterogeneous diseases driven by a multifactorial etiology involving genetic predisposition, hormones, and environmental factors; viruses are considered indisputable causal factors for nearly 10% of all human malignancies [2].

Human cytomegalovirus (HCMV), a member of the family Herpesviridae subfamily Betaherpesvirinae, is a ubiquitous host-restricted virus with a seroprevalence between 40% and 95% in the adult population [3]. Growing evidence suggests a link between persistent HCMV infection and malignancy [4,5,6,7]. Beyond oncomodulation, several studies implicate HCMV as a viral promoter of oncogenesis due to the presence of its nucleic acids and/or proteins in common tumor types [8,9]. Furthermore, HCMV promotes the acquisition of cancer hallmarks such as sustaining proliferative signaling, inducing angiogenesis, evading growth suppressors, avoiding immune destruction and apoptosis, facilitating replicative immortality, activating invasion, and metastasis as well as contributing to therapeutic resistance [8].

Recently, polyploidy, a major hallmark of cancer, was found to be present in approximately 37% of human tumors [10]. Dormant polyploid giant cancer cells (PGCCs) are associated with the induction of quiescence and increased storage capacity through the presence of vacuoles and accumulation of lipid droplets, increased metabolic capacity, and elevated energy production [11,12,13]. PGCCs could be triggered by radiotherapy, chemotherapeutics, hypoxia, oxidative stress, hyperthermia, and oncoviruses [14,15,16,17,18]. Beside therapy resistance, PGCCs were associated with metastasis and cancer relapse [18]. A recent study revealed the potential of HCMV in inducing the dedifferentiation of mature HMECs and generating PGCC’s phenotype as well as showing a significant correlation between the presence of PGCCs and HCMV in breast cancer biopsies [4].

Between 15% and 20% of all human cancers possess a direct infectious origin [19]. To start with, human papillomavirus (HPV) strains were classified into high- and low-risk strains after being isolated from different lesions. These discoveries were shown to radically alter the tumor diagnosis, prognosis, and prevention approaches [20,21]. Various Epstein–Barr virus (EBV) strains are associated with properties that influence their transforming capacities and the type of induced tumor [22,23]. Additionally, Kaposi’s sarcoma-associated herpesvirus (KSHV) isolates were identified in patients of different geographical regions, indicating the importance of these newly isolated strains [24,25] in developing better diagnostic procedures and novel treatment approaches in the context of KSHV-associated malignancies as well as enriching potential vaccine studies [26].

Triple-negative breast cancer (TNBC) comprises 15% of breast cancers globally [27]. Despite its susceptibility to standard chemotherapy, TNBC is highly invasive, has a high relapse tendency, and is associated with a poor overall prognosis [28]. Recently, more significant advances include characterizing the molecular features of TNBC which will maximize the efficacy of certain chemotherapeutic agents and aid in actively exploring novel therapeutic targets [29].

Previous studies demonstrated the ability of HCMV-DB and HCMV-BL strains in transforming primary HMECs into CMV-Transformed HMECs (CTH) cells in vitro [4,30]. CTH cells, which are slow self-renewing cells, undergo diverse stages of the giant cell cycle. They were shown to be heterogeneous, generate PGCCs, exhibit dedifferentiation, and display stemness and EMT/MET features [4]. Herein, we originally isolated two HCMV strains, B544 and B693, from TNBC biopsies to assess their transformation potential in vitro and evaluate the obtained cellular phenotype, the genetic and molecular features, and the activation of cellular stemness programs. We examined the sensitivity of these strains to paclitaxel (PTX) and ganciclovir (GCV) combination therapy. Further, a specific molecular landscape was disclosed in the tumor microenvironment (TME) of TNBC harboring high-risk HCMV.

## 2. Materials and Methods

### 2.1. Cell Lines and Culture

HMECs (A10565, Life Technologies, Carlsbad, CA, USA), CTH cells, MDA-MB231 as well as MCF7 (Institut Hiscia, Arlesheim, Switzerland), and MRC5 (RD-Biotech, Besançon, France) were cultured as previously described [5]. HCMV-B544 and HCMV-B693 cultures used in this study were maintained for at least 9 months in culture.

### 2.2. HCMV Isolates Growth and Detection

Data corresponding to TNBC biopsies and other HCMV strains were reported previously [5]. To prepare cell-free virus stocks, the two strains were propagated in MRC5 cells for a few passages to avoid losing the ULb’ region. Infections of HMECs and MRC5 cells, viral quantification, and replication including assessment of HCMV existence were performed as described previously [4]. Briefly, infection of HMECs or MRC5 (1 × 10^6^) cells with the clinical isolates was performed at a multiplicity of infection (MOI) of 1. Cells were incubated at 37 °C for two hours, after which the inoculum was discarded, and the cell monolayer was washed three times using 1X PBS and afterwards covered with fresh medium. For viral quantification, infectious supernatant (cell-free) was harvested, DNA was isolated (EZNA Blood DNA Kit, D3392-02, Omega BIO-TEK) and real-time IE1 quantitative PCR (qPCR) was carried out using a Stratagene Mx3005P thermocycler (Agilent, Santa Clara, CA, USA) and IE1 primers. qPCR was performed using a KAPA SYBR FAST Master Mix (KAPA BIOSYSTEMS, KK4601, Potters Bar, UK); reactions were activated at 95 °C for 10 min, followed by 50 cycles (15 s at 95 °C and 1 min at 60 °C). Results were collected and analyzed using MxPro qPCR software. The primers used are listed in Supplementary Table S1.

### 2.3. Microscopy

CTH cell cultures were monitored and confocal microscopy imaging for HMECs and CTH cells was performed as described previously [5,31]. Primary antibodies targeting IE1, pp65, Myc, Ki67 Ag, Oct4, Nanog, SOX2, and SSEA4 are listed in Supplementary Table S2. Post-staining, the slides were assessed using a 63× oil immersion objective lens with a Carl-Zeiss confocal microscope (Jena, Germany); images were analyzed using ZenBlue Software (Carl-Zeiss Microscopy GmbH).

### 2.4. Soft Agar Colony Formation Assay

Colony formation in soft agar seeded with uninfected HMECs, B544, B693, DB, and BL-infected HMECs, as well as untreated and PTX/GCV-treated CTH cells, was carried out as mentioned previously [5]. MCF7 and MDA-MB231 were used as positive controls.

### 2.5. Tumorsphere Assay

Tumorsphere formation by uninfected HMECs and HMECs infected with HCMV-B544 and HCMV-B693 was evaluated as described in detail previously [6].

### 2.6. Flow Cytometry Analysis

Cells (1 × 10^5^) were collected from uninfected HMECs and CTH cells, fixed, permeabilized, and stained as previously reported [5,6]. Cytofluorometric analysis was achieved using a BD LSRFortessa X-20 (BD Biosciences) flow cytometer. FACSDiva software (BD Biosciences) was used for data collection and analysis. Primary antibodies and their corresponding secondary antibodies are detailed in Supplementary Table S2.

### 2.7. Quantitative Reverse Transcription PCR (RT-qPCR)

Reverse transcription was performed as mentioned previously [6]. In brief, RNA was extracted from the biopsies using the EZNA^®^ Total RNA Kit I (Omega BIO-TEK, Norcross, GA, USA). Reverse transcription was carried out using the SuperScript IV First-Strand Synthesis kit (Invitrogen, Carlsbad, CA, USA). The expression of markers was assessed by performing real-time qPCR using a KAPA SYBR FAST Master mix (KAPA BIOSYSTEMS, KK4601) and specific primers according to the manufacturer’s protocol. The fold change expression was calculated by adopting the delta-delta Ct method. The primers used are provided in Supplementary Table S1.

### 2.8. CTH Treatment

CTH cells were treated with 12-O-45 tetradecanoylphorbol-13-acetate (TPA) (100 nM, P8139, Sigma-Aldrich, Burlington, MA, USA), ganciclovir (20 μM, SML2346 Sigma-Aldrich), and paclitaxel (20 nM, Paclitaxel Arrow 6 mg/mL). Treatment was renewed every day.

### 2.9. Statistical Analysis

All quantitative results are reported as mean ± SD of the independent experiments. Statistical analyses were performed using Mann–Whitney test; a *p*-value ≤ 0.05 was considered significant. Microsoft Excel was used to construct the plots and histogram data.

## 3. Results

### 3.1. Growth of Two HCMV Clinical Strains Isolated from TNBC in HMECs and the Emergence of Morphologically Distinct Cells

**Figure 1:**
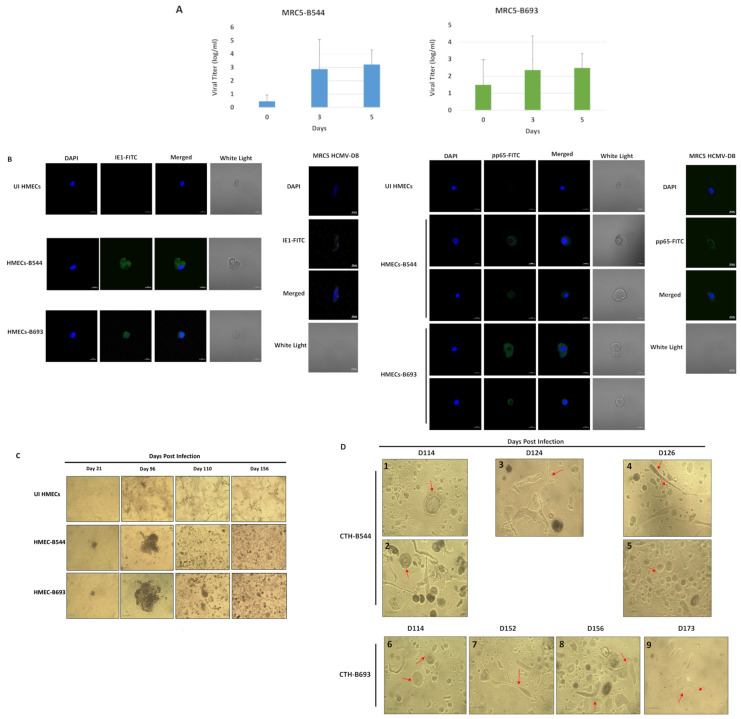
Replication of B544 and B693 strains in MRC5 cultures, and the appearance of morphologically distinct cells following the infection of HMECs with these high-risk strains. (**A**) Time-course of the viral titer in the supernatant of MRC5 infected with the strains HCMV-B544 and HCMV-B693, as measured by IE1-qPCR. (**B**) Confocal microscopic images of HCMV-IE1 and pp65 staining in HMECs infected with HCMV-B544 and HCMV-B693 (day 1 post-infection). Uninfected HMECs were used as controls. Nuclei were counterstained with DAPI; magnification ×63, scale bar 10 μm. (**C**) HMECs time-course infection with HCMV-B544 and HCMV-B693 strains (MOI = 1). Magnification ×100, scale bar 100 μm. Uninfected HMECs were used as a control. (**D**) Presence of giant cells with blastomere-like morphology (1 and 6), mesenchymal cells (4 and 7), lipid droplet-packed cells (3, 8, and 9), cells displaying multiple nuclei (2) as well as cell budding (4, 5, and 6), and cells with filopodia protrusions (9) in CTH-B544 and CTH-B693 cells. The inverted light microscope scale bar represents 100 µm; magnification ×200.

To assess the cellular environment achieved by HCMV, B544 and B693 were isolated from TNBC biopsies and grown in MRC5 cells, revealing a viral growth at days 3 and 5 post-infection (PI) (Figure 1A). At day 1 post-HMECs infection, HCMV-IE1 and pp65 were detected with confocal microscopy (Figure 1B). HCMV-B544 and B693 promoted the transformation of the infected HMECs toward CTH cells as previously reported [5]. Uninfected HMECs were used as controls which underwent cellular senescence in long-term cultures. At day 110 PI, we detected a wide variety of spheroids and giant cells distributed between round dense cells, flat, and elongated spindle-like cells. Afterwards, lipid droplet-packed cells, multinucleated giant cells, cell budding, and filopodium protrusions were observed in CTH cultures (Figure 1C,D). Hence, this population displayed mesenchymal and fibroblastic-like structures in addition to epithelial and small cells. The above-mentioned detailed cell morphology is close to that of CTH cells which were previously detected in HMEC cultures acutely infected with high-risk BL and DB strains [5]. Thus, the CTH-B544 and CTH-B693 heterogeneous cell population represent transformed and self-renewing cells that are engaged in distinct phases of the giant cell cycle [12,13].

### 3.2. Transformation Capacity of CTH Cells and the Induction of an Oncogenic Environment

**Figure 2:**
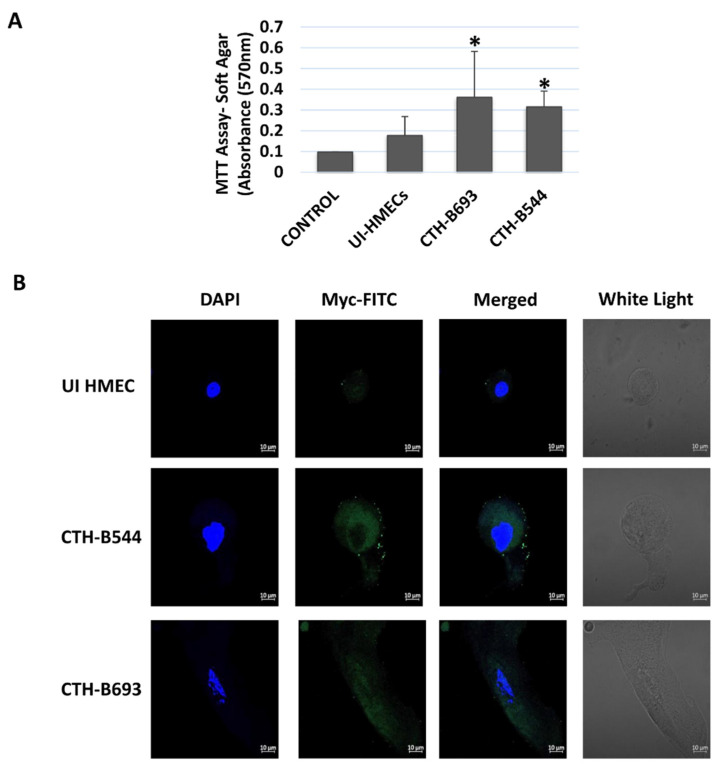
Colony formation in soft agar and Myc expression in CTH-B544 and B693 cells. (**A**) Colony formation in soft agar seeded with uninfected HMECs, CTH-B544 and CTH-B693 cells. At day 15 post-seeding, quantification of colonies was performed; Histogram represents the mean data ± SD of three independent experiments. * *p*-value ≤ 0.05, (**B**) Confocal microscopic images of Myc and DAPI staining in CTH cells. Magnification ×63, scale bar 10 μm.

**Figure 3:**
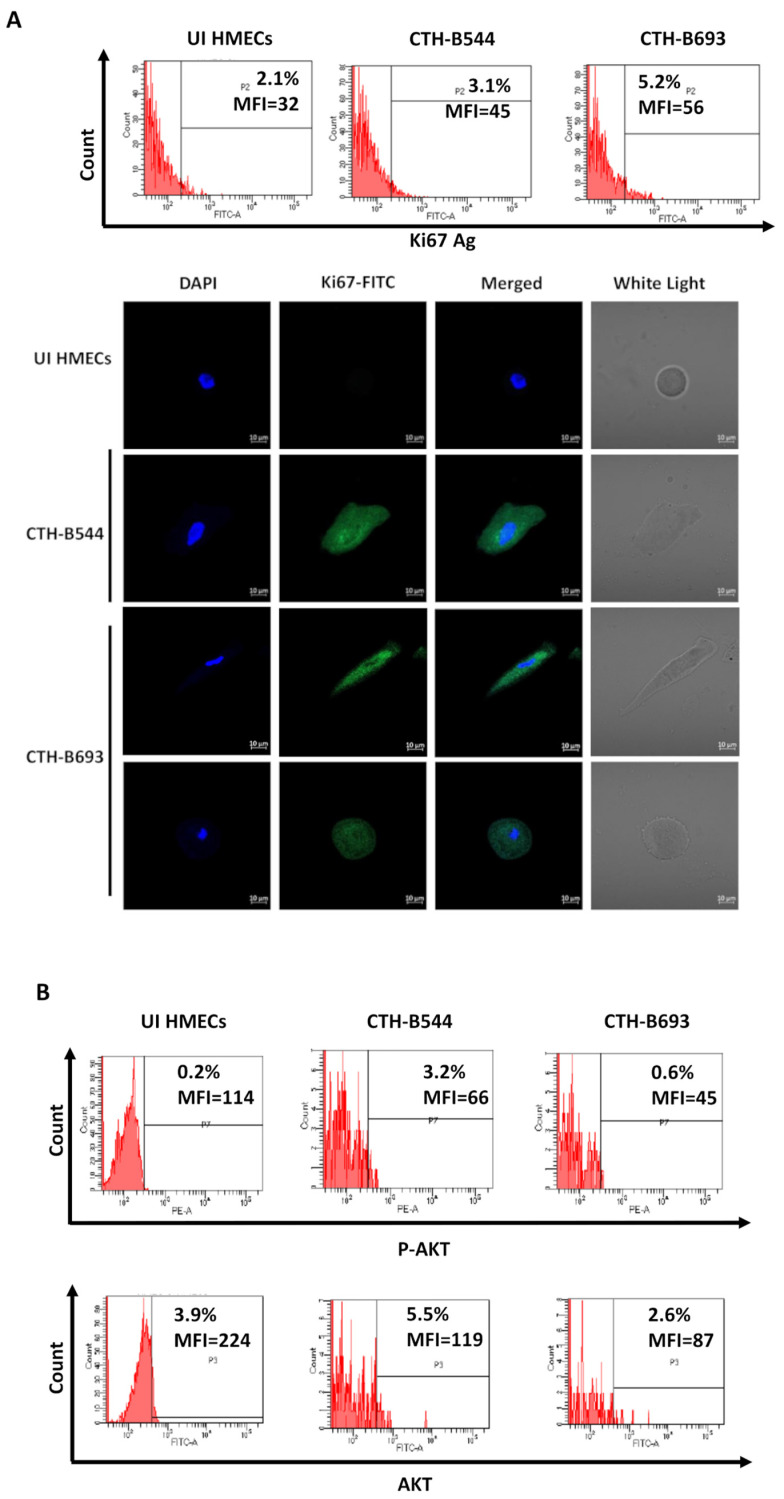
CTH proliferation capacities and AKT activation. (**A**) Proliferation assessment by FACS and confocal microscopy; UI HMECs, CTH-B544 and CTH-B693 cells were stained for Ki67 Ag and DAPI. Magnification ×63, scale bar 10 μm. (**B**) P-AKT, and AKT expression in UI HMECs and CTH cells, as measured by FACS. Results are representative of three independent experiments.

To evaluate the transformation of HCMV-B544 and B693 immortalized infected HMECs, cells were seeded in soft agar. Colony formation was detected at day 14 post-seeding in CTH-B544 and CTH-B693 cells (*p*-value ≤ 0.05) in contrast to uninfected HMECs which showed no changes (Figure 2A). The resulting anchorage-independent growth in CTH cells is a crucial phase in the acquisition of malignancies. With regard to oncogenes, the expression of c-Myc was assessed by performing confocal microscopy imaging where large and elongated CTH cells showed remarkable c-Myc staining compared to uninfected HMECs (Figure 2B). Using flow cytometry, a slightly higher expression of the proliferation marker (Ki67Ag) was detected in CTH-B693 compared to CTH-B544 cells with a limited expression in the uninfected HMECs. Only CTH-B544 and B693 cells were positively stained with Ki67 Ag as detected by confocal microscopy imaging (Figure 3A). On the other hand, a slight increase in the expression of phosphorylated Akt (pAkt-ser473) along with a limited to nonexistent increase in Akt expression levels was shown in all CTH cells compared to uninfected HMECs (Figure 3B and Supplementary Figure S1). Overall, the acquisition of an immortal phenotype in CTH-B544 and B693 cells reflects their transformation potential.

### 3.3. CTH Cells Promote Embryonic Stemness and Develop an Epithelial/Mesenchymal Hybrid State

**Figure 4:**
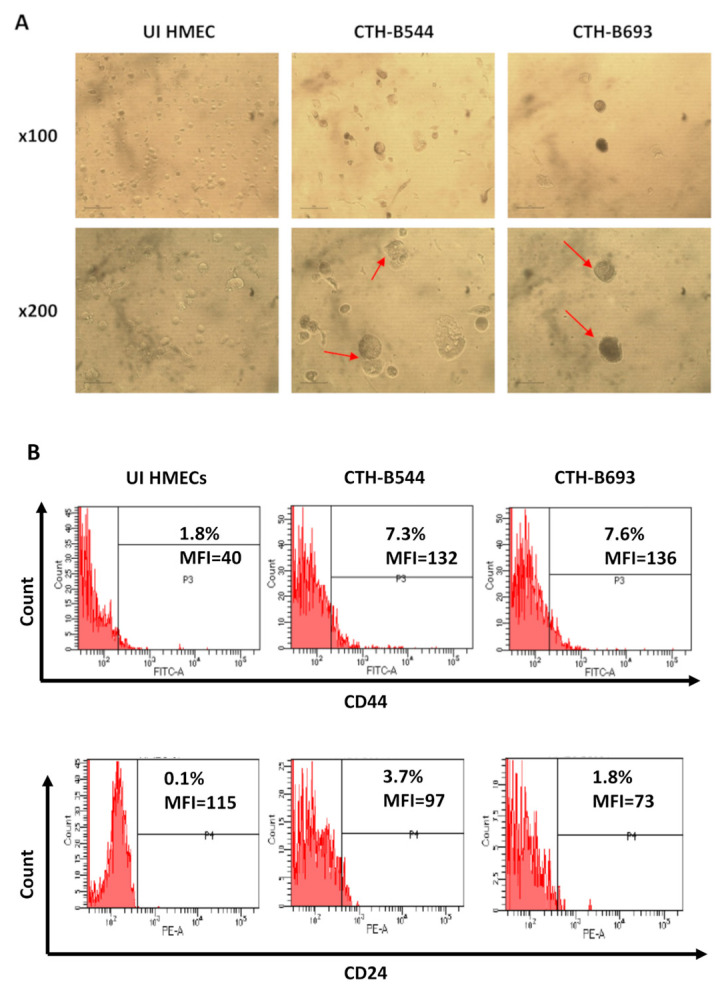
Tumorspheres formation and the expression of stemness markers in CTH cells. (**A**) Tumorspheres were observed under an inverted light microscope in CTH-B544 and CTH-B693 cells. Magnification ×100 and ×200, scale bare 100 µm. Uninfected HMECs were used as a negative control. (**B**) FACS staining of CD44 and CD24 was performed in CTH cells. Results are representative of three independent experiments.

**Figure 5:**
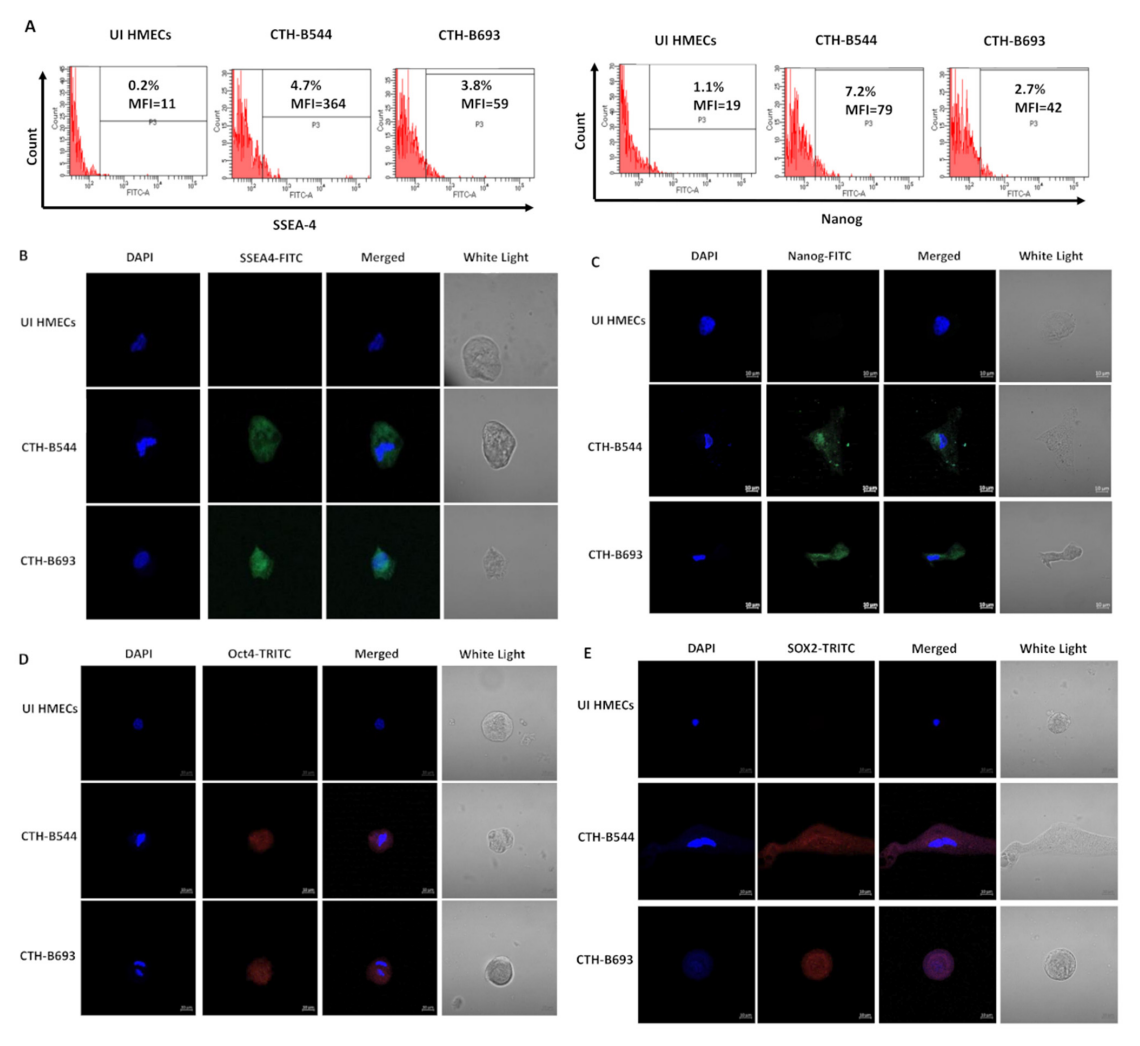
Expression of embryonic stem cell markers in CTH-B544 and B693 cells. (**A**) Detection of SSEA4 and Nanog in CTH cells by FACS. Results are representative of three independent experiments. Confocal microscopy imaging demonstrating the expression of (**B**) SSEA-4, (**C**) Nanog, (**D**) Oct4, and (**E**) SOX2. Nuclei were counterstained with DAPI; magnification ×63, scale bar 10 μm.

**Figure 6:**
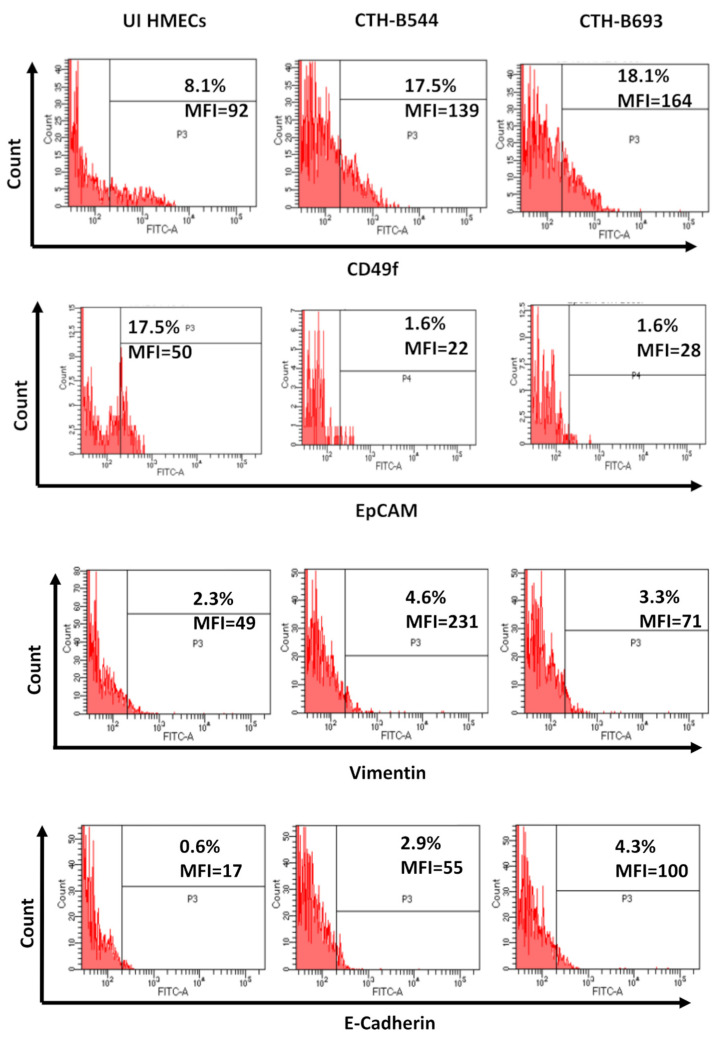
Phenotypic analysis of CTH cells. Detection of a panel of cell markers through FACS staining of CD49f, EpCAM, Vimentin, and E-cadherin in CTH-B544 and CTH-B693 versus UI HMECs. Results are representative of three independent experiments.

When cultured in serum-free tumorsphere medium, CTH-B544 and B693 cells gave rise to mammospheres at day 14 and expressed stemness markers versus uninfected HMECs (Figure 4A). CTH cells revealed a rise in CD44 and CD24 expression when compared to uninfected HMECs (Figure 4B), in line with CTH-DB and BL data (Supplementary Figure S1). Activated expression of the embryonic stem cell markers, SSEA-4 and Nanog, was recognized in CTH-B544, CTH-B693, CTH-DB, and CTH-BL cells by performing flow cytometry (Figure 5A and Supplementary Figure S1) and confocal microscopy imaging (Figure 5B,C). Moreover, CTH-B544 and B693 cells gained embryonic stem-like properties by highly expressing Oct4 and SOX2 as demonstrated by confocal microscopy imaging (Figure 5D,E). Similar to CTH-DB and BL, the newly discovered CTH cells showed an elevated expression of the stemness marker CD49f or Integrin alpha-6, and a limited EpCAM expression (Figure 6 and Supplementary Figure S1). CTH-B544 and B693 as well as CTH-DB and BL cells were positive for both vimentin and E-cadherin staining (Figure 6 and Supplementary Figure S1) thereby signifying their ability to dynamically oscillate between the epithelial-hybrid-mesenchymal spectrum as reported previously in tumors with poor prognosis [32,33]. As a result, CTH-B544 and B693 cells exhibited the following two phenotypes: stemness and hybrid epithelial/mesenchymal phenotypes.

### 3.4. Persistent HCMV Replication in CTH-B544 and CTH-B693 Long-Term Cultures

**Figure 7:**
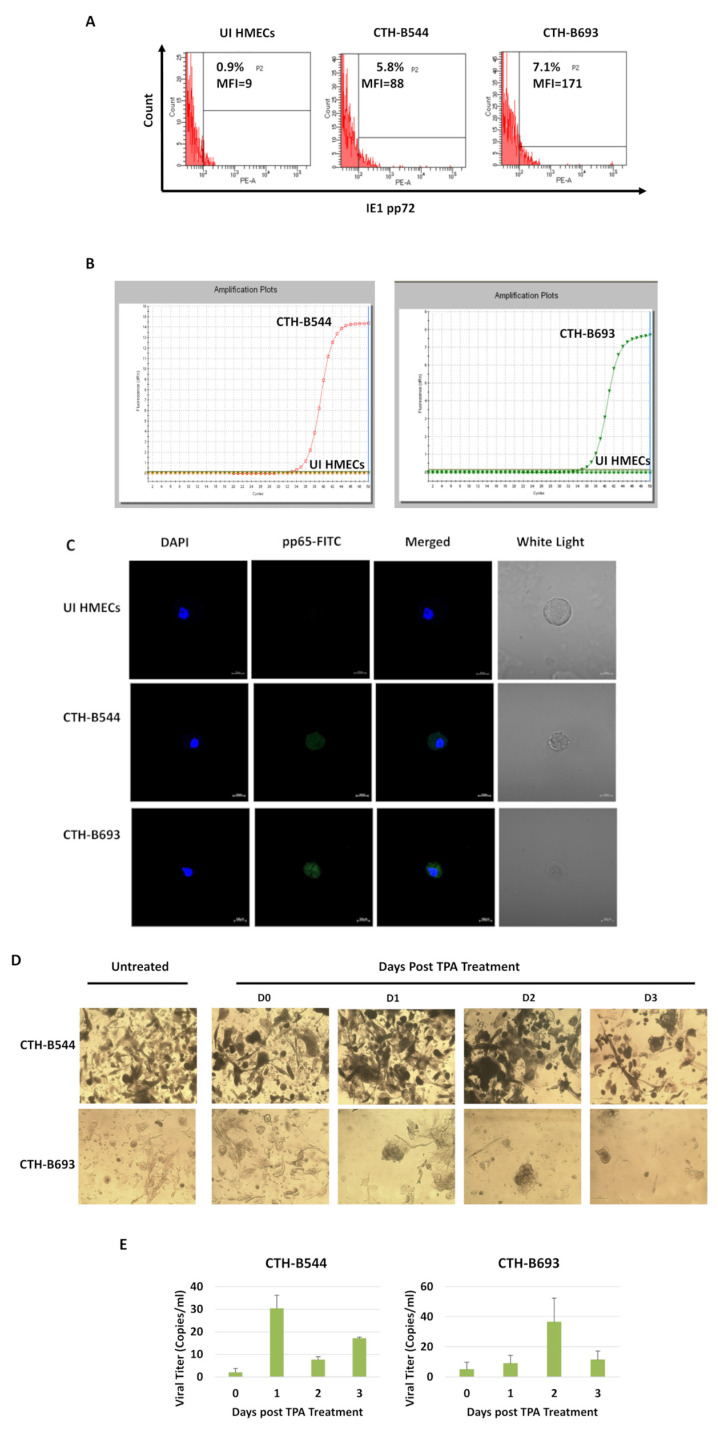
Sustained viral replication in CTH cells. IE1 expression in CTH cells was assessed by (**A**) FACS and (**B**) qPCR. (**C**) pp65 detection in CTH-B544 and B693 cells as demonstrated by confocal microscopy imaging. As a negative control, uninfected HMECs were used; nuclei were counterstained with DAPI; magnification ×63, scale bar 10 μm. (**D**,**E**) Determination of viral reactivation from latency in CTH cells through the treatment of TPA. (**D**) Representative images of CTH cultures treated with TPA (100 nM). Untreated cells were used as a control. Magnification ×100, scale bar 100 μm. (**E**) HCMV lytic replication was induced by TPA in CTH cultures. Histograms represent the mean data ± SD of three independent experiments.

To determine the sustained HCMV presence, HCMV-IE1 antigen was detected using flow cytometric analysis. IE1 was strongly expressed in CTH-B544 and CTH-B693; uninfected HMECs showed no staining for IE1 (Figure 7A). Using qPCR, we detected HCMV (IE1 DNA) in the supernatant of CTH-B544 and CTH-B693 cultures (Figure 7B). Further, CTH cells were positively stained for HCMV-pp65 antigen versus uninfected HMECs (Figure 7C). CTH cells were treated with TPA to assess latency relevance (Figure 7D,E). Post TPA treatment, the proliferation of CTH-B544 and CTH-B693 cells was promoted (Figure 7D). IE1 detection was elevated at day 1 and day 2 post-treatment in CTH-B544 and CTH-B693, respectively (*p*-value= 0.33), and subsequently decreased (Figure 7E).

### 3.5. A Specific Molecular Landscape Unveiled in the Tumor Microenvironment of TNBC Harboring High-Risk HCMV

**Figure 8:**
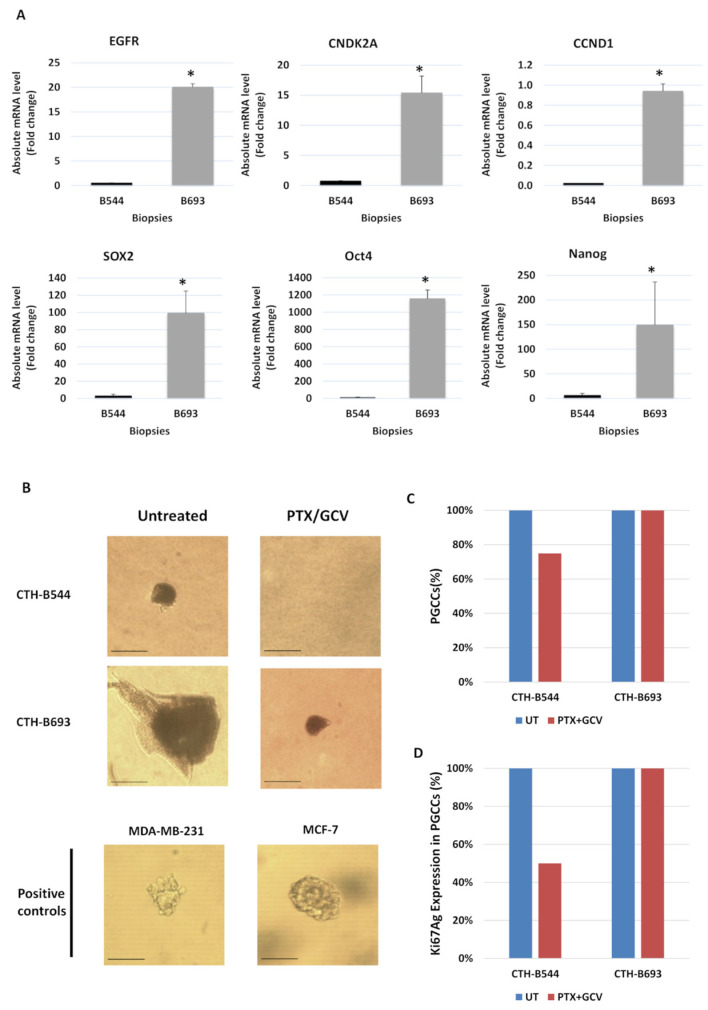
Distinct responses of CTH cells to paclitaxel/ganciclovir (PTX/GCV) treatment in vitro recapitulates distinct TNBC molecular signatures in vivo. (**A**) EGFR, CNDK2A, CCND1, SOX2, Oct4, and Nanog mRNA expression was measured by RT-qPCR in TNBC biopsies B544 and B693. Histograms represent the mean ± SD of three independent experiments. * *p* ≤ 0.05, determined by Mann–Whitney U test. (**B**) Soft agar seeded with CTH-B544 and CTH-B693 cells treated with PTX (20 nM)/GCV (20 μM) combination therapy. Untreated cells were used as negative controls; MCF7 and MDA-MB231 cells were used as positive controls. Magnification ×200, scale bar 100 μm. (**C**) Propidium iodide (PI) staining for detection of PGCCs in untreated and treated CTH cells by FACS analysis. (**D**) Ki67 Ag expression in PGGCs of untreated and treated CTH cells.

To evaluate the variation in strains’ aggressiveness, we compared the transcriptomic profile corresponding to the two high-risk biopsies. The absolute mRNA level of epidermal growth factor receptor (EGFR), cyclin dependent kinase inhibitor 2A (CNDK2A), cyclin D1 (CCND1), SOX2, Oct4, and Nanog was assessed by RT-qPCR. EGFR, a proto-oncogene that enhances cell proliferation and survival, the cell cycle regulator CNDK2A, the proliferation marker CCND1, and the three embryonic markers were overexpressed in biopsy 693 compared to biopsy 544 (*p*-value ≤ 0.05 for all markers) (Figure 8A). Our results indicated that biopsy 693, from which we isolated the HCMV-B693 strain, is associated with higher tumor aggressiveness and poor prognosis.

### 3.6. Restricting Soft Agar Colony Formation, Controlling PGCCs Count and Proliferation by Paclitaxel and Ganciclovir Therapy

To assess the colony formation in soft agar, CTH cells were treated by PTX/GCV combination therapy to target the oncogenic cellular environment as well as HCMV. Breast cancer cell lines, MDAMB231 and MCF7 were used as positive controls. PTX/GCV treatment of CTH-B544 cells resulted in the disappearance of colonies; however, colony formation was restricted in treated CTH-B693 cells versus untreated cells (Figure 8B). Post therapy, the PGCCs count was reduced by 25% in CTH-B544 while it remained constant in CTH-B693 as measured by FACS (Figure 8C). Further, the proliferation of PGCCs was assessed by Ki67 Ag measurement using flow cytometric analysis; Ki67 Ag expression was reduced in PTX/GCV-treated CTH-B544, but not in CTH-B693 (Figure 8D). Our outcomes revealed that CTH-B544 cells are more responsive to PTX/GCV therapy compared to CTH-B693 cells implying that the latter exhibits aggressive behavior.

## 4. Discussion

To the best of our knowledge, the present study demonstrated the oncogenic transformation and stemness potential of HCMV-B544 and B693 strains that were isolated from TNBC biopsies and indicated a differential treatment response depending on the HCMV strain present in the tumor. 

CTH-B544 and CTH-B693 cells are heterogeneous cellular populations that give rise to PGCCs, and display dedifferentiating phenotypes with stemness features as well as hybrid epithelial/mesenchymal phenotypes, resembling the morphological features found in aggressive cancers [13,14,17]. PGCCs formation was promoted in hypoxic environments [12,17]. It is worth noting that hypoxia inducible factor 1 alpha (HIF-1α) expression is induced by HCMV infection [34]. In PGCCs, the evaluation of metabolic reprogramming revealed the presence of PLIN4, a perilipin covering the lipid droplets especially in chemo-resistant tumors [13]; the Warburg effect [35] and the involvement of the glycolytic pathway were also found to be induced in cancer environments and upon HCMV infection [12].

Myc activation has been widely described in breast cancer progression and can be used as a predictive marker for cancer staging, therapy resistance, and prognosis [36]. It is noteworthy that both B544 and B693 HCMV strains were isolated from EZH2^High^Myc^High^ -expressing TNBC, further [5] indicating a potential link between the presence of these HCMV strains and cancer progression. The coupling of c-Myc overexpression with Akt pathway activation observed in CTH cells is in line with previous findings [4]. Moreover, colony formation was detected in CTH-B544 and CTH-B693 cells associated with a high expression level of Ki67 Ag and c-Myc overexpression, thus revealing cellular transformation and their oncogenic potential which is consistent with the previously reported CTH data [4]. Ki67, a prognostic biomarker in invasive breast cancer, is not only required for cell proliferation in tumors but is also strongly linked to tumor initiation, growth, and metastasis [37].

PGCC-bearing CTH cells acquired embryonic-like stemness and an epithelial-mesenchymal hybrid phenotype. Studies have shown that the acquisition of the EMT and stemness properties lead to an increase in the invasiveness and the metastatic potential of cancer cells within tumors [32]. The embryonic stem cells transcriptional network is based on the presence of master pluripotency regulators, Oct4, SSEA-4, SOX2, and Nanog [38]. Besides mammosphere generation, CTH-B544 and CTH-B693 gained a stemness phenotype with increased expression of Oct4, SOX2, and Nanog promoting tumor progression; CTH cells were positively stained for SSEA4 which is associated with EMT and drug resistance [38]. SOX2, Nanog, and Oct4 expression was associated with poor differentiation, advanced cancer stages, and the worst outcomes in breast cancer patients [39].

Studies have implicated CD44 in breast cancer cell adhesion, proliferation, motility and migration, angiogenesis, and metastasis. Limited CD24 expression in breast cancer cells was shown to augment their growth and metastatic potential through a chemokine receptor response [38]. The CD44high/CD24low phenotype was recognized in CTH-B544 and B693 cells indicating a tumor-initiating phenotype similar to that of the highly tumorigenic breast cancer cells [40]. The existence of an intermediate state between epithelial and mesenchymal phenotypes is considered a hybrid E/M state which is associated with elevated cellular plasticity, migration, stem-cell-like properties, metastatic potential, and therapy resistance [32]. In CTH cells, the co-existence of vimentin and E-cadherin resembled a partial EMT hence ensuring their plasticity while preserving the same tumor-propagating potential [4]. Since in breast tumors CD49f was considered a marker for distant metastasis and recurrence, CD49f+/CD44high/CD24low CTH cells represented an aggressive phenotype which is associated with an increased risk for disease recurrence with poor clinical outcomes [41]. 

The initiation of KSHV and EBV lytic cycles has been shown to support malignancies driven by the aforementioned oncogenic viruses [42,43]. HCMV persistence was established in CTH cells by detecting IE1 and pp65 throughout long-term cultures [4,5,30]. High-risk strains express immediate-early (IE), early (E), and late (L) viral antigens including IE1 in agreement with a viral lytic cycle following the acute infection of permissive cells such as MCR5 cells. High-risk strains are detected in chronically infected cells, for instance CTH cells in our study, which is in line with the HCMV latency observed in Hodgkin’s disease and Non-Hodgkin’s lymphoma revealing the latent viral UL138 protein expression [44]. Nonetheless, a dynamic state of latency is recommended by novel transcriptomic studies. Since HCMV develops a complex relationship with the host, to define lytic and latency phases, several studies used the ratio of replicative and latency genes as a phase indicator [45,46]. To further highlight the role of the HCMV-IE1 gene, a study showed the potential of HCMV in regulating stemness in glioblastoma cells by specifically increasing SOX2 and Nestin, thus upregulating stemness and proliferation markers [47].

Studies have suggested that the polymorphism of oncoviruses’ strains plays a vital role in explaining their association with distinct pathogenic and tumor properties [22,42]. Certain HPV strains were isolated from invasive cervical cancer biopsies and have thus been acknowledged as oncogenic/high-risk HPV strains [42]. EBV strains cloned and rescued from nasopharyngeal carcinoma (NPC) and gastric carcinomas potentially infect epithelial cells compared to strains originating from infectious mononucleosis (IM) or B lymphoma [22,48]. The existence of diverse strains expressing potential biomarkers and possessing various replication potentials in different tissues and/or cell types explain the distinct oncogenicity [22,23]. The variability of oncogenic and pathogenic KSHV potential was indicated to be dependent on KSHV different subtypes [42]. Overall, discovering distinct viral strains will adjust the different adopted approaches to tumor diagnosis, prognosis, prevention, advanced treatment, as well as therapy monitoring and will enrich potential vaccine studies [21,26,49]. Likewise, the diversity of HCMV strains was validated by our data and the previously published data in which only few clinical HCMV strains conserve the potential to transform HMECs [6] and fit with a “blastomere-like” model of oncogenesis [4]. When HCMV-B544 and B693 were isolated from IE1/Myc/Ki67/EZH2-positive TNBC biopsies [5], the findings revealed a phenotype similar to the already described HCMV-DB and BL phenotype. 

On the transcriptomic level, biopsy 693 overexpressed EGFR, CNDK2A, CCND1, SOX2, Oct4, and Nanog. It is known that EGFR promotes TNBC progression through JAK/STAT3 signaling [50], CNDK2A drives TNBC tumorigenesis [51], CCND1 has a prognostic significance in TNBC [52], and the three embryonic markers correlate with stemness, metastasis, tumor relapse, and poor clinical outcomes of TNBC [53]; therefore, we propose that biopsy 693 maintains a tumor signature that is associated with an aggressive behavior which predicts poor clinical outcome.

A study revealed the potential of combining GCV with certain chemotherapeutic agents to suppress EBV-positive NPC tumor growth [54]. In HPV-infected cervical cancer cells, cidofovir and cisplatin inhibited cellular proliferation, reduced E6 protein expression, and restored the activity of p53 [55]. A third study showed the effectiveness of anti-herpetic drugs, GCV and cidofovir, as single therapies or in combination with chemotherapy in treating KSHV-associated primary effusion lymphoma (PEL) [56]. Based on our results, the heterogeneity of HCMV strains including their distinct behavioral aspects had a major impact on CTH cells’ response post-therapy. CTH-B544 cells were therapy sensitive whereas CTH-B693 cells displayed an aggressive behavior with lower sensitivity to PTX/GCV combination therapy. Generally, isolating distinct HCMV strains from tumors that possess potential prognostic biomarkers and behave differently depending on their own heterogeneity and various cell types may improve the diagnostic process and treatment options, provide effective follow-up strategies, and may be essentially pertinent in breast cancer pathophysiology and other adenocarcinomas, particularly of poor prognosis.

## 5. Conclusions

Our outcomes originally revealed the oncogenic and stemness potential of two HCMV strains, namely B544 and B693 where HCMV generated PGCCs, displayed dedifferentiating phenotypes with stemness features as well as hybrid epithelial/mesenchymal phenotypes. Large-scale experiments are highly encouraged to further validate our findings. Meanwhile, the presented data provides new insights into the oncogenic role of HCMV in breast cancer progression, thereby uncovering novel targeted therapeutic approaches and assisting in the development of advanced clinical interventions.

## Data Availability

The datasets used and/or analyzed during the present study are available from the corresponding author on reasonable request.

## Supplementary Materials

The following supporting information can be downloaded at: https://www.mdpi.com/article/10.3390/cancers14174271/s1, Supplementary Figure S1: Activation of oncogenic pathways, expression of embryonic markers, and phenotypic characterization of CTH cells. Flow cytometric analysis of CTH-DB cells, CTH-BL cells, MRC5 cells infected with HCMV-DB, and MRC5 cells infected with HCMV-BL for (A) Ki67 (B) pAKT and AKT, (C) CD44 and CD24, (D) SSEA4 and Nanog, (E) CD49f, vimentin, E-cadherin, and EpCAM, and (F) IE1. Results are representative of three independent experiments; Supplementary Table S1: List of Primers Used; Supplementary Table S2: List of Antibodies Used.
