# Supplementary material for: Oncogenic and Stemness Signatures of the High-Risk HCMV Strains in Breast Cancer Progression
Source: Cancers (Basel). 2022 Sep 1;14(17):4271. doi: 10.3390/cancers14174271 (PMC9455011; doi:10.3390/cancers14174271)
Supplement: Supplementary file 1 [file cancers-14-04271-s001.zip › Supplementary Figure S1.pdf]

Supplementary Data

A

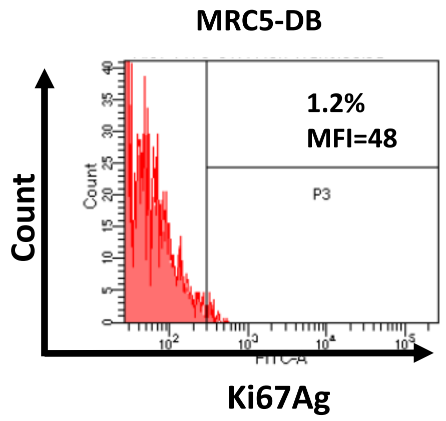

B

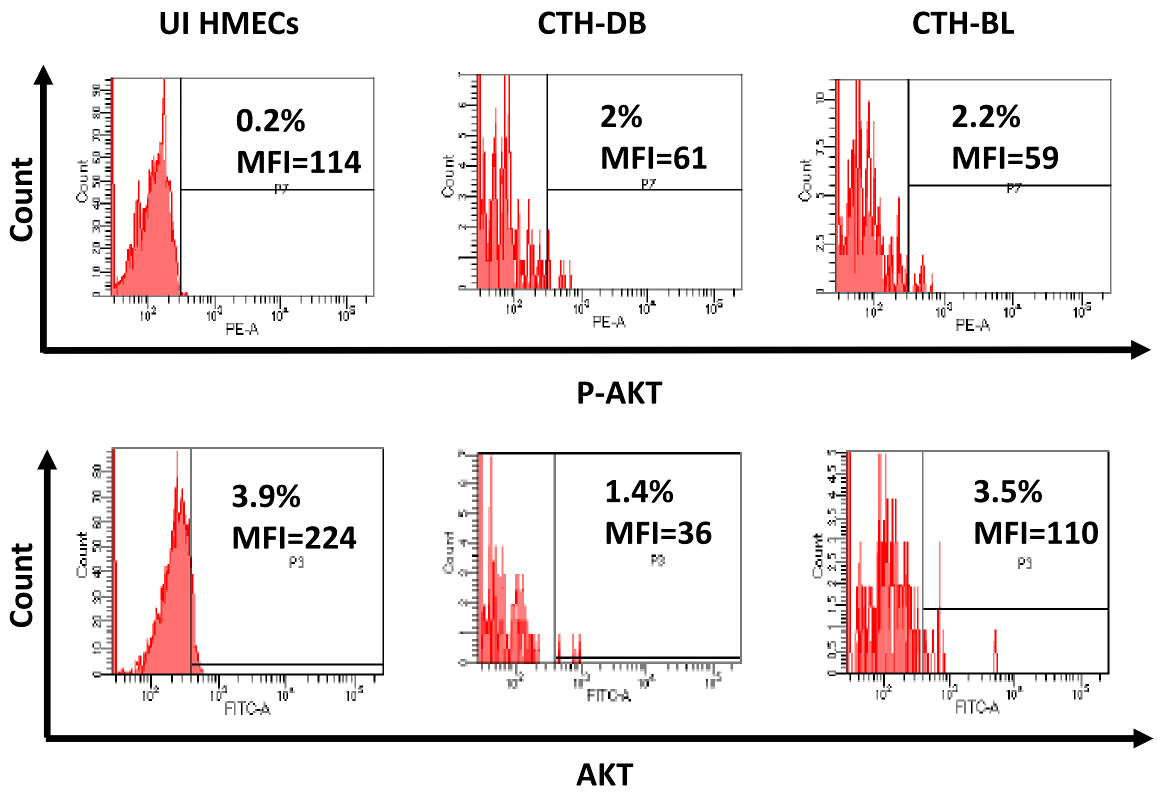

UI HMECs
CTH-DB
CTH-BL
Count
0.2%
MFI=114
2%
MFI=61
2.2%
MFI=59
PE-A
P-AKT
3.9%
MFI=224
1.4%
MFI=36
3.5%
MFI=110
FITC-A
AKT

C

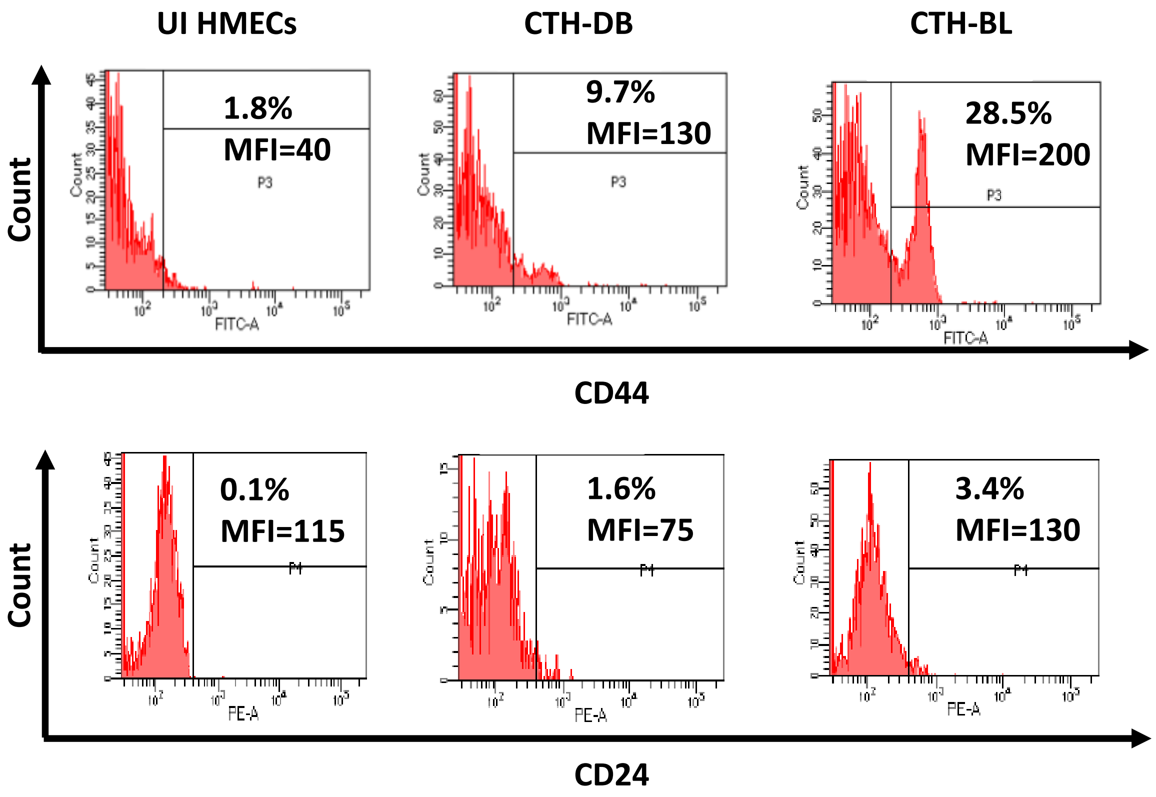
UI HMECs
CTH-DB
CTH-BL
Count
1.8%
MFI=40
9.7%
MFI=130
28.5%
MFI=200
CD44
Count
0.1%
MFI=115
1.6%
MFI=75
3.4%
MFI=130
CD24

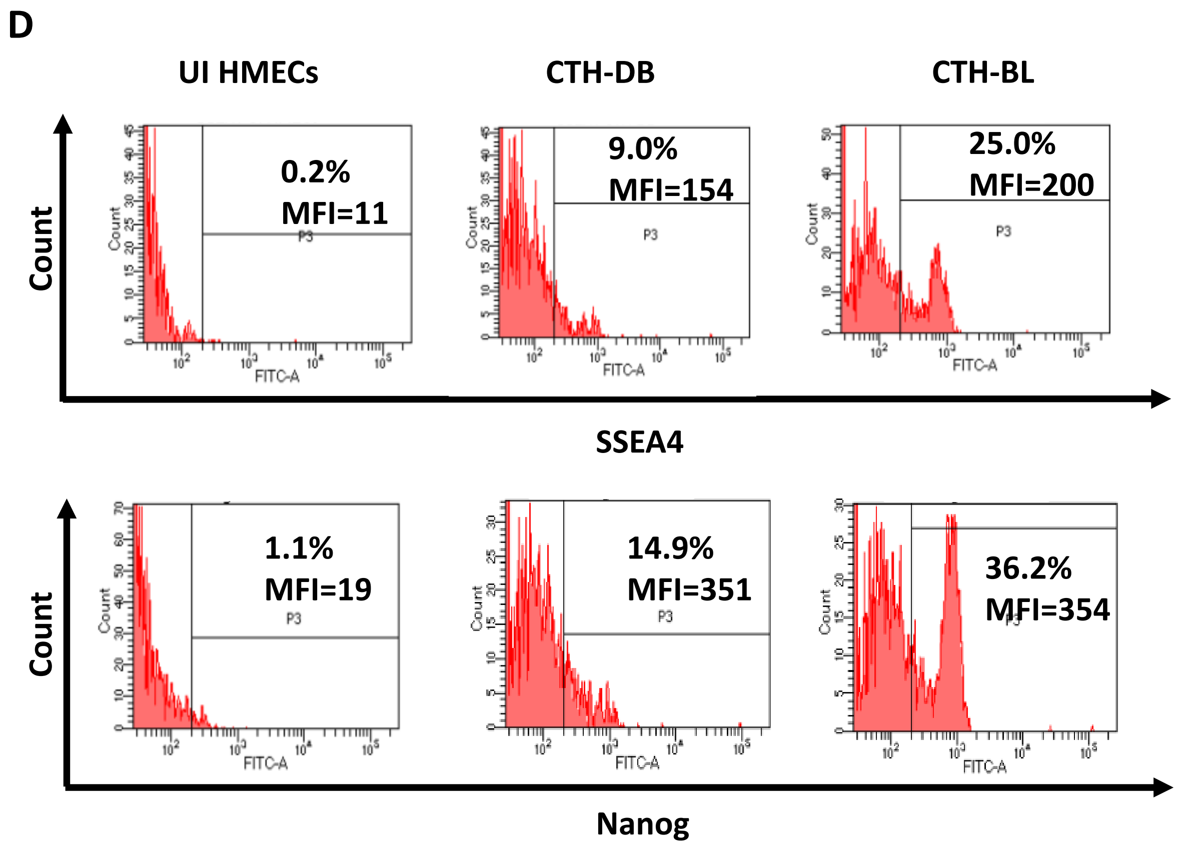
D
UI HMECs
CTH-DB
CTH-BL
Count
0.2%
MFI=11
9.0%
MFI=154
25.0%
MFI=200
P3
FITC-A
SSEA4
1.1%
MFI=19
14.9%
MFI=351
36.2%
MFI=354
Nanog

E

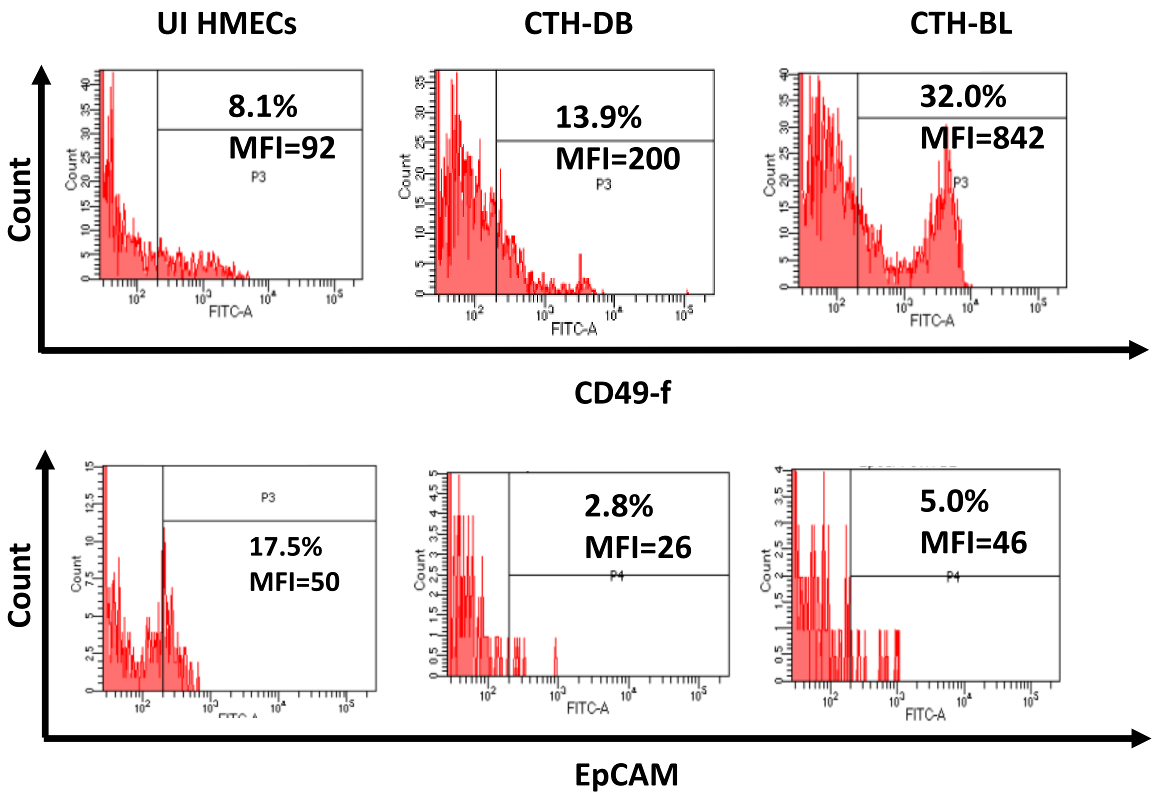
UI HMECs
CTH-DB
CTH-BL
Count
8.1%
MFI=92
13.9%
MFI=200
32.0%
MFI=842
CD49-f
Count
17.5%
MFI=50
2.8%
MFI=26
5.0%
MFI=46
EpCAM

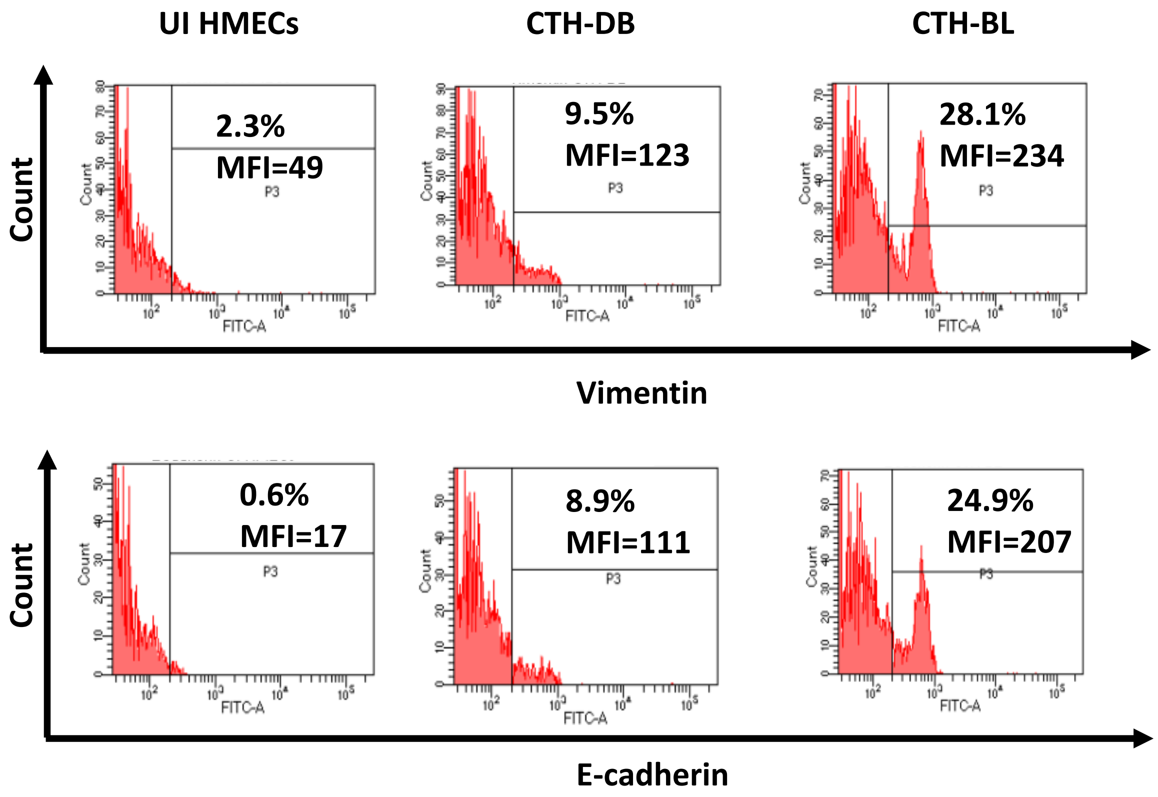
UI HMECs
CTH-DB
CTH-BL
Count
2.3%
MFI=49
9.5%
MFI=123
28.1%
MFI=234
Vimentin
Count
0.6%
MFI=17
8.9%
MFI=111
24.9%
MFI=207
E-cadherin
FITC-A
P3

F

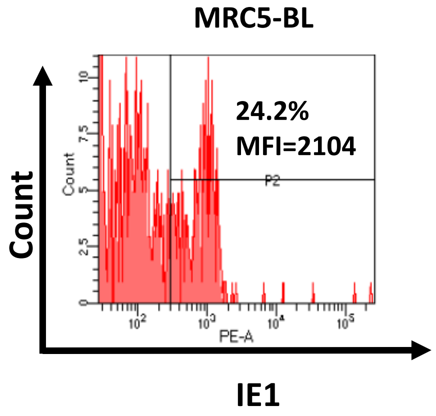

Supplementary Figure S1: Activation of oncogenic pathways, expression of embryonic markers, and phenotypic characterization of CTH cells. Flow cytometric analysis of CTH-DB cells, CTH-BL cells, MRC5 cells infected with HCMV-DB, and MRC5 cells infected with HCMV-BL for (A) Ki67 (B) pAKT and AKT, (C) CD44 and CD24, (D) SSEA4 and Nanog, (E) CD49f, vimentin, E-cadherin, and EpCAM, and (F) IE1. Results are representative of three independent experiments.
