# Supplementary material for: Oncogenic and Stemness Signatures of the High-Risk HCMV Strains in Breast Cancer Progression
Source: Cancers (Basel). 2022 Sep 1;14(17):4271. doi: 10.3390/cancers14174271 (PMC9455011; doi:10.3390/cancers14174271)
Supplement: Supplementary file 1 [file cancers-14-04271-s001.zip › Supplementary Tables.pdf]

Supplementary Table S1. List of Primers Used.

Primer	Primer Sequence
IE1-forward	5'-CGACGTTCCTGCAGACTATG-3'
IE1-reverse	5'-TCCTCGGTCACTTGTTCAAA-3'
Nanog-forward	5′-TCCTCCTCTTCCTCTATACTAAC-3′
Nanog-reverse	5′-CCC ACAATCACAGGCATAG-3′
SOX2-forward	5′-GGGAAATGGAGG GGTGCAAAAGAGG-3′
SOX2-reverse	5′-TTGCGTGAGTGT GGATGG GATTGGTG-3′
Oct4-forward	5′-TGGAGAAGGAGAAGCTGGAGCAAAA-3′
Oct4-reverse	5′-GGCAGAGGTCGTTTGGCTGAATAGACC-3′
CNDK2A-forward	5′-GGGAGCAGCATGGAGCCG-3′
CNDK2A-reverse	5′-AGTCGCCCGCCATCCCCT-3′
CCND1-forward	5′-GCGAGGAACAGAAGTGC-3′
CCND1-reverse	5′-GAGTTGTCGGTGTAGATGC-3′
EGFR-forward	5′-TGCGTCTCTTGCCGGAAT-3′
EGFR-reverse	5′- GGCTCACCCTCCAGAAGGTT-3′
GAPDH-forward	5′-CCCCTCTTCAAGGCCTCTAC-3′
GAPDH-reverse	5′-CGACCACTTTGTCAAGCTCA-3′

Supplementary Table S2. List of Antibodies Used.

Antibody	Catalog Number/Source
Myc	06-549/Upstate, Lake Placid, NY, USA
Ki67	BD-556026/BD Biosciences (Franklin Lakes, USA)
CMV pp72 (IE1)	SC-69834/Santa Cruz Biotechnology (CA, USA)
IE1	ab53495/Abcam (Cambridge, UK)
Oct4	ab19857/Abcam (Cambridge, UK)
Nanog	SC-293121/Santa Cruz Biotechnology (CA, USA)
SOX2	ab97959/Abcam (Cambridge, UK)
EpCAM	BD-347197/BD Biosciences (Franklin Lakes, USA)
pp65	SC-58116/Santa Cruz Biotechnology (CA, USA)
SSEA-4	SC-21704/Santa Cruz Biotechnology (CA, USA)
AKT	SC-5298/Santa Cruz Biotechnology (CA, USA)
pAKT (Ser473)	SC-293125/Santa Cruz Biotechnology (CA, USA)
CD24	BD-555428/BD Biosciences (Franklin Lakes, USA)
CD44	BD-555478/BD Biosciences (Franklin Lakes, USA)
CD49f	BD-555735/BD Biosciences (Franklin Lakes, USA)
Vimentin	SC-6260/Santa Cruz Biotechnology (CA, USA)
E-cadherin	SC-7870/Santa Cruz Biotechnology (CA, USA)
FITC-conjugated anti-mouse antibody	BD- 553399/BD Biosciences (Franklin Lakes, USA)
PE-conjugated anti-mouse antibody	BD-551436/BD Biosciences (Franklin Lakes, USA)
FITC-conjugated anti-rabbit antibody	ab6717/Abcam (Cambridge, UK)
FITC-conjugated Goat Anti-Mouse	BD-555988/BD Biosciences (Franklin Lakes, USA)
FITC-conjugated Rat Anti-Mouse	BD-553443/BD Biosciences (Franklin Lakes, USA)
